# Clinical software development for the Web: lessons learned from the BOADICEA project

**DOI:** 10.1186/1472-6947-12-30

**Published:** 2012-04-10

**Authors:** Alex P Cunningham, Antonis C Antoniou, Douglas F Easton

**Affiliations:** 1 Centre for Cancer Genetic Epidemiology, Department of Public Health and Primary Care, University of Cambridge, Strangeways Research Laboratory, Worts Causeway, Cambridge CB1 8RN, UK

**Keywords:** Clinical software development, Open source, Web, BOADICEA risk model

## Abstract

**Background:**

In the past 20 years, society has witnessed the following landmark scientific advances: (i) the sequencing of the human genome, (ii) the distribution of software by the open source movement, and (iii) the invention of the World Wide Web. Together, these advances have provided a new impetus for clinical software development: developers now translate the products of human genomic research into clinical software tools; they use open-source programs to build them; and they use the Web to deliver them. Whilst this open-source component-based approach has undoubtedly made clinical software development easier, clinical software projects are still hampered by problems that traditionally accompany the software process. This study describes the development of the BOADICEA Web Application, a computer program used by clinical geneticists to assess risks to patients with a family history of breast and ovarian cancer. The key challenge of the BOADICEA Web Application project was to deliver a program that was safe, secure and easy for healthcare professionals to use. We focus on the software process, problems faced, and lessons learned. Our key objectives are: (i) to highlight key clinical software development issues; (ii) to demonstrate how software engineering tools and techniques can facilitate clinical software development for the benefit of individuals who lack software engineering expertise; and (iii) to provide a clinical software development case report that can be used as a basis for discussion at the start of future projects.

**Results:**

We developed the BOADICEA Web Application using an evolutionary software process. Our approach to Web implementation was conservative and we used conventional software engineering tools and techniques. The principal software development activities were: requirements, design, implementation, testing, documentation and maintenance. The BOADICEA Web Application has now been widely adopted by clinical geneticists and researchers. BOADICEA Web Application version 1 was released for general use in November 2007. By May 2010, we had > 1200 registered users based in the UK, USA, Canada, South America, Europe, Africa, Middle East, SE Asia, Australia and New Zealand.

**Conclusions:**

We found that an evolutionary software process was effective when we developed the BOADICEA Web Application. The key clinical software development issues identified during the BOADICEA Web Application project were: software reliability, Web security, clinical data protection and user feedback.

## Background

In the past 20 years, society has witnessed the following landmark advances in the biological and computer sciences: (i) the sequencing of the human genome, (ii) the development and distribution of software by the open source movement, and (iii) the invention of the World Wide Web. The sequencing of the human genome [1,2] was a defining achievement of late 20th century science which had profound implications for our understanding of the genetic risk of disease. The work of the open source movement may be viewed as a cultural, scientific and computing phenomenon [3] which has had innumerable benefits for science and society. Similarly, the invention of the Web server [4], the implementation of Common Gateway Interface (CGI) programs, and the widespread adoption of open technological standards such as the Extensible Markup Language [5] have revolutionised the distribution and processing of digital information. Together, these advances have provided a new impetus for clinical software (CS) development: software developers now translate the products of human genomic research into CS tools; they use open-source programs to build them; and they use the Web to deliver them. These activities form the basis of many translational research projects. Whilst this open-source component-based approach has undoubtedly made CS development easier, CS projects are still hampered by long-standing problems that traditionally accompany the software process.

### This study

This study describes the development of the BOADICEA Web Application (BWA) [6], a computer program used by clinical geneticists to assess risks to patients with a family history of breast and ovarian cancer. The key challenge of the BWA project was to deliver a program within an acceptable timeframe that was safe, secure and easy for healthcare professionals to use. We focus on the BWA software process, problems faced, and lessons learned, so that other software developers can learn from our experience. Many problems described here are widespread in the software industry, and so our discussion has implications for other areas of software development.

The BWA project began in January 2005. By that time, some alternative genetic risk models had already been implemented as desktop applications (e.g. the BRCAPRO and Claus models implemented in CancerGene [7], and the Tyrer-Cuzick model implemented in IBIS [8]). However, the BWA was the first program of its kind to be made available on the Web.

#### Objectives

Our key objectives are: (i) to highlight key CS development issues; (ii) to demonstrate how software engineering tools and techniques can facilitate CS development for the benefit of individuals who lack software engineering expertise; and (iii) to provide a CS development case report that can be used as a basis for discussion at the start of future projects.

## Implementation

In this section, we describe the BOADICEA model, and the BWA project and software process.

### BOADICEA model

The Breast and Ovarian Analysis of Disease Incidence and Carrier Estimation Algorithm (BOADICEA) [9,10] is a risk model for familial breast and ovarian cancer. The model can be used to compute BRCA1 and BRCA2 mutation carrier probabilities and age specific risks of developing breast and ovarian cancer, using explicit family history data (pedigrees), age information, cancer diagnoses in family members, and BRCA1 and BRCA2 genetic testing information. The algorithm was developed using complex segregation analysis of breast and ovarian cancer based on a combination of families identified through population based studies of breast cancer, and families with multiple affected individuals who had been screened for BRCA1 and BRCA2 mutations. BOADICEA models the simultaneous effects of BRCA1 and BRCA2 mutations and assumes that the residual familial clustering of breast cancer is explained by a polygenic component (a large number of genes each of small effect), with a variance that decreases linearly with age. Individuals are assumed to follow calendar period and cohort specific incidence rates for breast and ovarian cancer. The BOADICEA model has been assessed [11] using data from UK genetics clinics and has been found to be well calibrated within these families and to discriminate well between BRCA1, BRCA2 and non-mutation carriers.

### BOADICEA Web Application project

BOADICEA was originally implemented as a standalone Fortran program (termed here the BOADICEA core program, BCP). The BCP has been used by scientists for some years as a research tool. However, in practice, computing risks with the BCP was difficult and time consuming, which made it inappropriate for use in a clinical setting. To address this problem, we developed the BWA, a user-friendly Web interface to the BCP which has greatly simplified this process. BWA v1 was released for general use 2 years and 10 months after the start of the project. The BWA project milestones were as follows: (i) BWA project begins (January 2005); (ii) BWA v1 trial software released for evaluation and testing (January 2007); (iii) BWA v1 released for general use (November 2007); (iv) BWA v2 trial software released for evaluation and testing (November 2009); and (v) BWA v2 released for general use (August 2010). This work took longer than anticipated, but there were important mitigating factors: (i) none of our team had any prior experience of CS development for the Web; (ii) our list of requirements was substantial (see Requirements section below); and (iii) the development of the online pedigree building module was particularly difficult and time consuming (see Design section below).

### Software process

**Figure 1:**
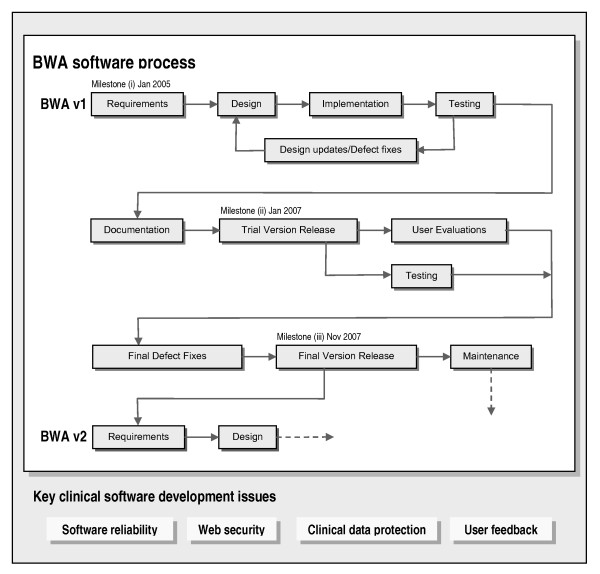
**BOADICEA Web Application software process**. This flowchart shows the main activities in the BWA software process described in the text (project milestones are labelled). The key clinical software development issues identified during the project are listed at the bottom of the figure.

We developed the BWA using an evolutionary software process (Figure 1). Our approach to Web implementation was conservative and we used conventional software engineering tools and techniques. We did not conform strictly to an established software process model. However, the BWA software process was iterative and program modules were delivered in increments, and so there were similarities with the incremental process model (e.g. Pressman [12]). The principal software development activities were: requirements, design, implementation, testing, documentation and maintenance. These activities are described below.

#### Requirements

At the start of the BWA project, we established the functional requirements of the program. In particular, the program would enable the user to: (i) build new pedigree data sets quickly and flexibly online; (ii) upload pre-existing pedigree data sets; (iii) review the pedigree data set in a table and drawing; (iv) compute BRCA1 and BRCA2 mutation carrier probabilities and breast/ovarian cancer risks; (v) view the computed results in data tables; and (vi) download the input pedigree data set and computed results in plain text and PDF format. These requirements were set out in a software requirements specification document with mock-ups of the Web interface.

The software requirements specification document ensured that requirements were properly understood, and that problematic design issues were considered from the outset. Clinical geneticists were involved in the requirements process from the start of the BWA project. Their advice helped to ensure as far as possible that the program would be easy to use in a clinical setting. We believe that the involvement of end users in the requirements process was key to the success of the project. Owing to the complexity of the proposed software, we could not anticipate all program behaviours at the outset. As a result, Web interface designs evolved substantially as the project progressed. Since the release of BWA v1, software development has been driven by two further ongoing requirements: (i) the need to accommodate extensions to the BOADICEA model; and (ii) the need to implement suggestions for improvement made by users.

#### Design

Our main objectives were to design a Web interface that would: (i) have a look and feel that would seem familiar to clinical geneticists; (ii) make risk calculations quick and easy; (iii) reduce data errors by constraining user inputs; (iv) function intuitively and respond intelligently to user inputs; (v) have a clear and consistent layout; (vi) have unambiguous data prompts; (vii) minimise key strokes and enable users to set defaults automatically; and (viii) help clinical geneticists to communicate results to patients (e.g. by providing a processing report PDF). The Web interface was also designed to process cached Web pages intelligently.

Our original aim was to design a Web interface that would make BOADICEA easy to use for clinical geneticists, researchers and members of the public. However, these individuals represented a wide spectrum of users with differing prior knowledge and expertise. As a result, we found it difficult to design a Web interface that could accommodate the diverse needs of all these different user groups. One alternative would have been to implement different modes of operation e.g. 'clinician mode' and 'public mode'. Patients could have used a simplified 'public mode' interface to build a preliminary pedigree data set prior to a consultation with a clinical geneticist. This would have helped to shift some of the burden of data entry from the counsellor to the counselee. However, time and funding constraints prevented us from designing two separate modes of operation. As a result, our main aim was to design a program that would be easy to use in a clinical setting.

The online pedigree building module was intended to enable users to build pedigrees quickly and easily. However, in practice, the module delivered with BWA v1 was difficult and time consuming to use. This problem was principally one of Web interface design. Work on this module was problematic because: (i) it included pedigree building functions that were previously unavailable in comparable Web-based programs, and so required a novel design solution; and (ii) we were hampered in this task by our lack of prior Web programming experience. After further development, the pedigree building module delivered with BWA v2 did fulfil all initial requirements. This was made possible by refinements to the design of the interface. Our experience of this task was consistent with the observation that 'the incompletenesses and inconsistencies of our ideas become clear only during implementation' [13], or that 'you often don't really understand the problem until after the first time you implement a solution' [3].

#### Implementation

The BCP had been implemented in Fortran prior to the start of the BWA project. We chose to implement the BWA CGI module in Perl for the following reasons: (i) Perl was a de facto language for CGI programming; (ii) the CGI.pm module [14] and Perl regular expressions would make handling Web form data easier; (iii) Perl text processing functions could be used to create and manipulate the many data files required by the BCP; (iv) Perl could be used to run all necessary external programs and to implement separate software installation and backup scripts; (v) Perl taint mode would help to make the program more secure; and (vi) the Comprehensive Perl Archive Network [15] would provide numerous open-source modules to interface with databases and other software. In addition, we used JavaScript to control Web page behaviours and to validate Web form parameters in the user's browser.

We also made extensive use of open-source technologies including the Linux operating system, Apache Web server, MySQL database, Perl interpreter, gfortran compiler, R statistical computing package, Kinship pedigree drawing package, ImageMagick image processing package, html2ps document converter, Concurrent Versions System version control package and Xemacs programming editor.

We used a bottom-up development scheme: we developed low level modules first, and then combined them to form higher level ones. We also used the following defensive programming techniques [16]: (i) we used assertions; (ii) we checked the values of input from external sources; (iii) we handled exceptions gracefully; (iv) we implemented subroutines with low coupling to try to contain the damage caused by errors; and (v) we checked function return values. The source code included hundreds of assertions to check for data processing errors. When an assertion failed during program execution, we modified the code to ensure that it would not fail again in the same way. The assertions also made it easier to identify new defects introduced during source code modifications.

When we implemented the BWA, our aim was to write source code that was stable, reliable, maintainable and extensible. We attempted to write source code that could be easily understood by others, as this helped to make clear both the behaviour of the program and the underlying intentions of the programmer. At the start of the project, we established in-house coding conventions according to good practice [16] and enforced them. Wherever possible, source codes were broken into cohesive modules and stored in libraries. For clarity, we tried to separate JavaScript and HTML as far as possible.

#### Testing

The aim of software testing was to find and eliminate defects (faults that failed on execution). Before the BWA was released, we ran an initial set of in-house tests and applied defect fixes and design updates on the basis of the test results. This process was iterative. After these initial tests, we wrote the accompanying documentation and released the BWA v1 trial software for evaluation by users (users were informed of its trial status). During this period of evaluation, we ran a further set of in-house tests. Once this further phase of in-house testing was complete and we had implemented the final defect fixes, we released BWA v1 for general use.

In-house software tests were planned and executed systematically, and test results were documented. We prioritised tests to exercise modules that had the potential to generate the most harmful defects. Test cases were designed to uncover specific classes of defect, and to ensure that data were displayed consistently. The most serious defects were uncovered by in-house tests. Some of these defects were due to simple semantic errors, or to errors of omission. This observation is sobering. However, we found that software faults could be easily overlooked when the program included several tens of thousands of lines of source code.

We considered software reliability to be the most important issue in BWA development. Consequently, nearly 30% of our development time was devoted to testing. We found that in-house tests and user evaluations were complementary as these activities were based on internal and external views of the program respectively. During the software evaluations, users provided essential feedback. As users developed a better understanding of the program, so we developed a better understanding of how they were using it. In this way, user feedback formed a vital part of the software process. Nevertheless, in spite of this, we recognised that users could only identify a subset of defects as: (i) user evaluations were not systematic; and (ii) users were only likely to identify a defect if a computed result (or software behaviour) differed significantly from their own expectation.

#### Documentation

BWA documentation consisted of the user guide [17] and frequently asked questions on the project Web site [6]. The user guide provided simple instructions on the use of the program, and described limitations of the BOADICEA model and key input data requirements. We always asked users to read the user guide before they used the program, but we had to accept that some individuals may not have had time to do so. The Cambridge University Legal Services Department also drafted a software license agreement that set out our terms of use.

#### Maintenance

The principal aims of software maintenance were to implement defect fixes and enhancements to the BWA, and to support the BWA user community. Web deployment made some maintenance tasks easier because: (i) we could update program executables on the server easily; (ii) we did not have to maintain different software builds for different operating systems; and (iii) we could log user activity on the server.

The installation of the BWA v2 trial software revealed a potential software maintenance pitfall. To implement this new software, we had to upgrade several open-source components (e.g. Perl, R and MySQL). However, we found that some of these updated components were incompatible with BWA v1. At that time, we already had > 900 users, and it was essential that these upgrades did not cause an interruption to service. To address this problem, we setup a separate Linux test server and ran a trial software installation to ensure that both BWA v1 and v2 worked together with the updated components. Once we had resolved the incompatibilities and the trial software installation was successful, we could then perform the same installation on our production server without problems.

## Results

### System description

**Figure 2:**
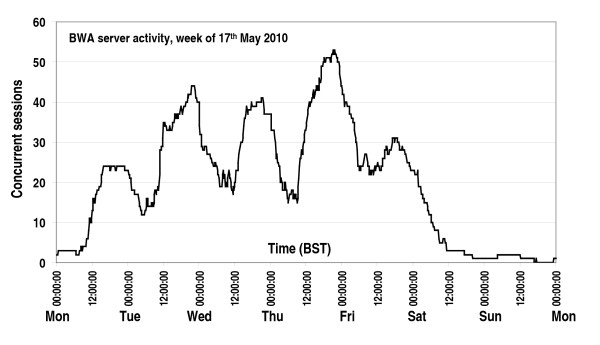
**BOADICEA Web Application server activity logged during the week of the 17th May 2010**. This curve shows the number of concurrent BWA sessions initiated on the server by users of BWA v1 and v2 trial software. The prominent peaks in the curve correspond to peak user activity during weekday evenings. Times plotted as the independent variable were recorded during British Summer Time.

Clinical geneticists use the BWA to calculate BRCA1 and BRCA2 mutation carrier probabilities and breast/ovarian cancer risks. These data are used to determine eligibility for genetic testing or to identify individuals at high risk of developing breast/ovarian cancer who can then be offered individually tailored clinical management. BWA v1 was released for general use in November 2007. By May 2010, we had > 1200 registered users based in the UK, USA, Canada, South America, Europe, Africa, Middle East, SE Asia, Australia and New Zealand. Figure 2 shows the number of concurrent BWA sessions logged on the server during the week of the 17th May 2010. BWA users included clinical geneticists, researchers and members of the public.

#### Online workflow

**Figure 3:**
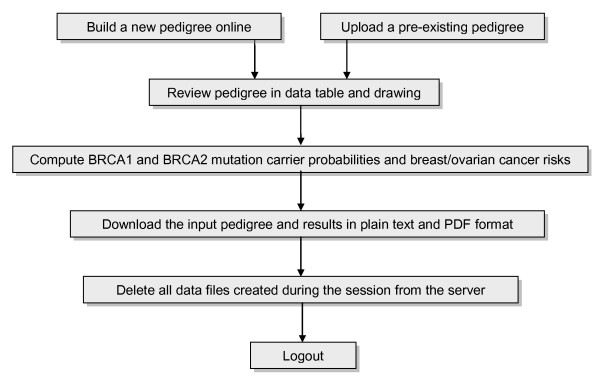
**BOADICEA Web Application workflow**. This flowchart shows the main processing steps in the online workflow described in the text.

To compute risks, users negotiate a simple online workflow (Figure 3). First, the user must supply an input pedigree data set. The user can either: (i) build a new pedigree data set online, or (ii) upload a pre-existing pedigree data set for processing. All clinical data are transmitted across an encrypted Web connection.

**Figure 4:**
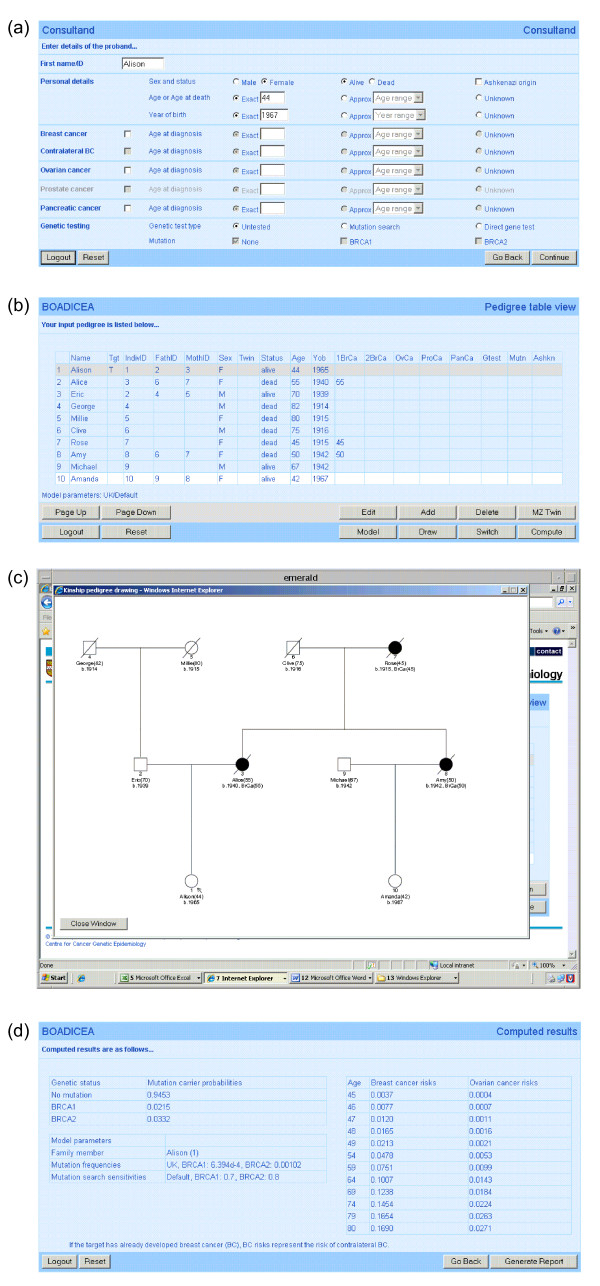
**(a) Family Member Web page**. Family Member Web pages are used to capture details of individual family members within the pedigree (in this case the consultand). Users are prompted for details of sex, vital status, Ashkenazi Jewish origin, age or age at death (age at last follow up), year of birth, cancer history and genetic status. Family Member Web pages have been designed so that users can input data quickly and easily. JavaScript functions control the behaviour of the input elements so that the Web page functions intuitively and responds intelligently to user inputs. JavaScript functions are also used to validate input data parameters when the Web page is submitted for processing. All data validations run on the Web page are repeated on the server for security purposes. **(b) Pedigree Table View Web page**. The Pedigree Table View Web page is used to display the tabulated input pedigree data set. Each table row includes details of a single family member, and each table column corresponds to a specific input data parameter. The Web page includes two rows of function buttons: the top row functions are used to navigate the table and edit the pedigree; the bottom row functions include functions to logout or reset the current session, to update BOADICEA model parameters, to draw the pedigree, to switch the target (index or subject of the risk calculation) and to compute risks. The target is highlighted in the table with a grey table row. **(c) Pedigree drawing**. Pedigree drawing generated using the Kinship package [21] implemented in the R environment [22]. The pedigree drawings are annotated in the conventional manner: the target is identified with an arrow, and family members who have developed cancer are shaded. The text annotation beneath each family member includes the following parameters: unique identifier, first name/ID and age or age at death (age at last follow up), year of birth, cancer history and genetic status. **(d) Computed Results Web page**. The Computed Results Web page lists BRCA1 and BRCA2 mutation carrier probabilities and breast/ovarian cancer risks computed by BOADICEA from the input pedigree data set (expressed as decimal probabilities). BRCA1 and BRCA2 mutation carrier probabilities are shown in the top left-hand table, and breast/ovarian cancer risks are shown in the right-hand table. The bottom left-hand table shows the BOADICEA model parameters used in the calculation.

To build a new pedigree data set online, the user first supplies details of the consultand and the consultand's parents on three 'Family Member' Web pages (Figure 4a). Once these details have been submitted, the program returns a 'Pedigree Table View' Web page (Figure 4b) listing these individuals in a table. The user can then use pedigree editing functions to add additional relatives, and to update individual data parameters.

Users can also upload a pre-existing pedigree data set for processing. To do this, the user must first create a pedigree data file in the BOADICEA import/export digital data format (a simple plain text format designed to facilitate pedigree data exchange, described in Appendix A of the user guide [17]). Some commercial pedigree data management/drawing programs such as Progeny [18], Clinical Pedigree [19] and PED [20] now include a BOADICEA data file export utility which has helped to widen access to BOADICEA further.

When the input pedigree data set is complete, the user can review it prior to running a risk calculation. The 'Pedigree Table View' Web page (Figure 4b) lists details of all family members in a data table. Similarly, the user can draw the pedigree using the Kinship package [21] implemented in the R environment [22] (Figure 4c).

Once the input pedigree data set has been finalised, the user can compute risks at the push of a button. The BWA returns BRCA1 and BRCA2 mutation carrier probabilities and age specific breast/ovarian cancer risks in a 'Computed Results' Web page (Figure 4d). The user can also adjust key model data parameters such as the population BRCA1 and BRCA2 mutation frequencies and mutation search sensitivities, so that the risk calculation can be tailored to different populations and genetic testing methods.

Once BOADICEA risks have been computed, the user can download the input pedigree data set and results in a plain text data file and processing report PDF. The PDF is intended to help genetic counsellors communicate risks to patients. Whilst the PDF includes tables of BRCA1 and BRCA2 mutation carrier probabilities and breast/ovarian cancer risks, the presentation of these data could be refined to facilitate communication further (e.g. breast and ovarian cancer risks could be plotted on a graph with equivalent population risks to aid their interpretation in a wider context). Ongoing research projects aimed at communicating risks effectively will inform further improvement of risk reporting.

Downloading the pedigree data set and storing it locally ensures that it can be updated at a later date [23]. Early in the BWA project, we considered the possibility of storing pedigree data sets on the BWA system. This would have enabled users to access their data from any location which would have made the program easier to use. However, this solution would also have conflicted with our requirement to conform to data protection principles (described in the Clinical data protection section below). Hence, the existing data storage solution may be viewed as a compromise intended to meet competing requirements for software usability and clinical data protection. When the user logs out, all data files created during the session are deleted from the server. Alternatively, if the user does not log out, the data files will be deleted after a period of 24 hours when the session times out.

The BWA has now made it much easier for clinical geneticists to run BOADICEA risk calculations in a clinical setting. As we develop the BWA further, we hope to achieve a better integration between the program and the wider genetic counselling workflow.

## Discussion

We found that an evolutionary software process was effective when we developed the BWA. The key CS development issues identified during the BWA project were: software reliability, Web security, clinical data protection and user feedback. These issues are described below.

### Key clinical software development issues

#### Software reliability

In our view, software reliability is the most important issue in CS development. Clinical geneticists use the BWA to calculate BRCA1 and BRCA2 mutation carrier probabilities and breast/ovarian cancer risks, which are used to determine eligibility for genetic testing or to provide individually tailored clinical management. As a result, defects in the program could have implications for the management of patients.

Software defects cause programs to function unreliably. Testing can help to find defects in substantial computer programs, but it is impossible to eliminate them completely (e.g. Myers [24] noted that 'in general, it is impractical, often impossible, to find all the errors in a program'). Hence, we have to accept that some defects are likely to remain undetected. McConnell [16] suggested that the 'industry average is about 15 to 50 errors per 1000 lines of code for delivered software'. BWA v1 included approximately 40000 lines of code. Consequently, we took steps to ensure as far as possible that the program functioned reliably before it was released for general use. In-house tests revealed the most serious defects. Less serious defects were reported by users during the software evaluations. As part of the software process, we enforced in-house coding rules and used defensive programming techniques [16]. We believe that these measures helped to prevent the introduction of faults and to make the software more reliable.

#### Web security

Web security is an additional challenge in CS development. All computers connected to the Internet are at risk of malicious attack, and Web servers running CGI applications are particularly vulnerable [25]. Consequently, we took steps to minimise the effects of a malicious attack should one occur. To help secure the BWA server, we ensured that: (i) clinical data were always stored outside the document root; (ii) appropriate permissions were set on program files; (iii) a firewall was in place; (iv) the operating system was patched; (v) non-essential services were shutdown; (vi) data transmissions were encrypted; (vii) Web form data were validated on the server before use; (viii) Perl components ran in taint mode; and (ix) computers that were detected probing the server were subject to access restrictions with Fail2ban [26]. The Cambridge University Computing Service also probed the server periodically to try to identify security vulnerabilities.

#### Clinical data protection

Clinical data protection is a further key issue in CS development. If healthcare professionals believe that a Web-based program may put patient data at risk, they will not use it. As a result, we took steps to conform as far as possible to the data protection principles set out in Schedule one to the Data Protection Act 1998 [27]: (i) the program does not use data items that are regarded as strong patient identifiers [28] (in particular, the program does not use full name [only first name is used which is optional], address, date of birth, postcode, NHS number or local patient identifier); (ii) we collected only the minimum data required to enable users to compute and interpret risks; and (iii) data were deleted when the user logged out, or after a period of 24 hours when the session timed out.

It is important to consider whether the identity of an individual could be inferred from the data submitted by users. The BWA prompts for only three of the 14 key items of patient-identifiable information set out in Appendix 7 of the Caldicott Committee Report [29]: forename (which is optional), sex and ethnic group. The Caldicott Committee Report states that 'an individual item from this list, taken with another item from a particular flow, may in certain circumstances enable identity to be inferred', and it gives as an example 'age linked to a diagnosis'. Since BWA users supply details of age and cancer diagnosis, it may be possible to infer an individual's identity from these data, or possibly from other combinations of data. However, in practice, the ease with which this can be accomplished depends on the nature of the data items and the context of their disclosure.

We also configured the BWA so that a user could only view data submitted during his/her current session. As a result, if for example an unauthorised person were to use John Smith's username and password to access the system, then any data sets submitted concurrently by John Smith would still be inaccessible to the unauthorised person, as they would be stored elsewhere.

#### User feedback

As soon as we released the BWA v1 trial software, we urged users to report computed results or software behaviours that differed significantly from their own expectation. Whenever a user queried a result, we first attempted to replicate their result using the BCP alone to determine whether the problem was associated with the BCP or the Web interface (results generated with the BCP had been compared with data from UK genetics clinics [11], but we were aware that the BCP could still include defects). We then sought to explain why the user's result failed to meet expectation. In most cases, users reported problems: (i) when the BOADICEA model behaved in a way that was unexpected to them (e.g. users were sometimes surprised by the effect that different mutation search sensitivities had on computed risks); and (ii) when key input data parameters were excluded unintentionally from the risk calculation. As a result, we learned that it was important to inform users of some specific BOADICEA model behaviours and key input data requirements. In this way, user inquiries formed the basis of many frequently asked questions on the project Web site [6]. By verifying user results, we gained further confidence that the BWA was functioning reliably.

## Conclusions

The BWA has now been widely adopted by clinical geneticists and researchers. Key observations and lessons learned during the BWA project were as follows:

1. We found that an evolutionary software process was effective when we developed the BWA. Our approach to Web implementation was conservative and we used conventional software engineering tools and techniques. The software process had to be sufficiently flexible to accommodate evolving Web interface designs. The key CS development issues identified during the BWA project were: software reliability, Web security, clinical data protection and user feedback.

2. The software requirements specification document ensured that requirements were properly understood, and that problematic design issues were considered from the outset.

3. We were unable to design a Web interface that would fully accommodate the diverse needs of clinical geneticists, researchers and members of the public. Our main aim was to design a program that would be easy to use in a clinical setting.

4. We had to devise a novel design solution for the online pedigree building module.

5. We implemented the BWA CGI module in Perl and made extensive use of open-source technologies.

6. In-house software tests were planned and executed systematically, and test results were documented. We prioritised tests to exercise modules that had the potential to generate the most harmful defects. The most serious defects were uncovered by in-house tests.

7. In our view, software reliability was the most important issue in CS development. Software testing helped us to find defects, but we had to accept that some defects were likely to remain undetected.

8. We took the following steps to improve software reliability: (i) we enforced in-house coding rules; (ii) we used defensive programming techniques; (iii) we tested the software extensively; and (iv) we urged users to report computed results and software behaviours that differed substantially from their own expectation.

9. We found that in-house tests and user evaluations were complementary as these activities were based on internal and external views of the program respectively. As users developed a better understanding of the program, so we developed a better understanding of how they were using it.

10. We took steps to minimise the effect of a malicious attack on the BWA server should one occur.

11. We took steps to conform as far as possible to the data protection principles set out in Schedule one to the Data Protection Act 1998.

12. We learned that it was important to inform users of some specific BOADICEA model behaviours and key input data requirements.

13. User feedback was extremely important to us and it helped to shape the software process. User inquiries formed the basis of many frequently asked questions on the project Web site. By verifying computed results queried by users, we gained further confidence that the BWA was functioning reliably.

## Availability and requirements

**Project name**: BOADICEA

**Project home page**: [http://www.srl.cam.ac.uk/genepi/boadicea/boadicea_home.html]

**Operating system**: The BWA is implemented on an Ubuntu Linux computer. Users access the software via the Web.

**Programming language**: The BWA is implemented in Perl and JavaScript, and the BCP is implemented in Fortran.

**Other requirements**: Modern Web browser with active scripting enabled

**License**: [http://www.srl.cam.ac.uk/genepi/boadicea/BWA_v2_Software_and_Data_Processing_Agreement.pdf]

## Abbreviations

CGI: Common gateway interface; CS: Clinical software; BWA: BOADICEA Web Application; BOADICEA: Breast and Ovarian Analysis of Disease Incidence and Carrier Estimation Algorithm; BCP: BOADICEA core program.

## Competing interests

The authors declare that they have no competing interests.

## Authors' contributions

APC implemented the BWA with guidance from his co-authors. ACA implemented the BOADICEA risk model. DFE and ACA managed the BOADICEA project and provided guidance on data processing issues, and software design and implementation. All authors read and approved the final manuscript.

## Pre-publication history

The pre-publication history for this paper can be accessed here:

http://www.biomedcentral.com/1472-6947/12/30/prepub
